# Supplementary material for: Machine Learning Accelerates Crystallization for Structure Determination
Source: Angew Chem Int Ed Engl. 2026 May 4;65(25):e1218503. doi: 10.1002/anie.1218503 (PMC13266958; doi:10.1002/anie.1218503)

checkCIF/PLATON report

Structure factors have been supplied for datablock(s) lcz099

THIS REPORT IS FOR GUIDANCE ONLY. IF USED AS PART OF A REVIEW PROCEDURE FOR PUBLICATION, IT SHOULD NOT REPLACE THE EXPERTISE OF AN EXPERIENCED CRYSTALLOGRAPHIC REFEREE.

No syntax errors found. CIF dictionary Interpreting this report

Datablock: lcz099

```
Bond precision:   C-C = 0.0087 A          Wavelength=1.54184

Cell:              a=12.6002(1)      b=14.7573(2)      c=42.4666(4)
                   alpha=90          beta=90           gamma=90
Temperature:       100 K

                 Calculated                  Reported
Volume           7896.45(14)                 7896.45(14)
Space group      P b c a                     P b c a
Hall group       -P 2ac 2ab                  -P 2ac 2ab
Moiety formula   C15 H3 Ag3 F18 N6, C26 H16  C15 H3 Ag3 F18 N6, C26 H16
Sum formula      C41 H19 Ag3 F18 N6          C41 H19 Ag3 F18 N6
Mr               1261.23                     1261.23
Dx,g cm-3        2.122                       2.122
Z                8                           8
Mu (mm-1)        12.998                      12.998
F000             4880.0                      4880.0
F000'            4901.72
h,k,lmax         16,18,53                    15,18,53
Nref             8490                        8244
Tmin,Tmax        0.141,0.273                 0.357,1.000
Tmin'            0.039

Correction method= # Reported T Limits: Tmin=0.357 Tmax=1.000
AbsCorr = MULTI-SCAN
```

Data completeness= 0.971 Theta(max)= 78.530

R(reflections)= 0.0651(7336) wR2(reflections)= 0.1636(8244)

S = 1.050 Npar= 641

The following ALERTS were generated. Each ALERT has the format
test-name_ALERT_alert-type_alert-level.
Click on the hyperlinks for more details of the test.

Alert level C

```
PLAT213_ALERT_2_C Atom F00I            has ADP max/min Ratio .....        3.8 prolat
PLAT213_ALERT_2_C Atom F00O            has ADP max/min Ratio .....        4.0 prolat
PLAT213_ALERT_2_C Atom F01Y            has ADP max/min Ratio .....        3.8 prolat
PLAT213_ALERT_2_C Atom F01U            has ADP max/min Ratio .....        3.5 prolat
PLAT213_ALERT_2_C Atom F01W            has ADP max/min Ratio .....        3.6 prolat
PLAT213_ALERT_2_C Atom F01X            has ADP max/min Ratio .....        3.2 prolat
PLAT342_ALERT_3_C Low Bond Precision on  C-C Bonds ...............    0.00872 Ang.
PLAT410_ALERT_2_C Short Intra H...H Contact  H01K      ..H01R     .       1.95 Ang.
                                                     x,y,z  =      1_555 Check
PLAT906_ALERT_3_C Large K Value in the Analysis of Variance ......      4.384 Check
PLAT911_ALERT_3_C Missing FCF Refl Between Thmin & STh/L=    0.600          6 Report
                4  5  0,   2 15  0,   0 16  0,   2 15  1,   4  0 20,   0  8 27,
PLAT934_ALERT_3_C Number of (Iobs-Icalc)/Sigma(W) > 10 Outliers ..          1 Check
               15  2  1,
PLAT972_ALERT_2_C Check Calcd Resid. Dens.  0.81Ang From Ag01          -2.16 eA-3
PLAT972_ALERT_2_C Check Calcd Resid. Dens.  1.69Ang From Ag01          -2.00 eA-3
PLAT972_ALERT_2_C Check Calcd Resid. Dens.  0.76Ang From Ag03          -1.88 eA-3
PLAT972_ALERT_2_C Check Calcd Resid. Dens.  0.77Ang From N00K          -1.88 eA-3
PLAT972_ALERT_2_C Check Calcd Resid. Dens.  0.90Ang From Ag03          -1.86 eA-3
PLAT972_ALERT_2_C Check Calcd Resid. Dens.  0.82Ang From Ag02          -1.79 eA-3
PLAT972_ALERT_2_C Check Calcd Resid. Dens.  0.73Ang From Ag01          -1.77 eA-3
PLAT972_ALERT_2_C Check Calcd Resid. Dens.  0.76Ang From Ag02          -1.76 eA-3
PLAT972_ALERT_2_C Check Calcd Resid. Dens.  0.96Ang From Ag02          -1.75 eA-3
PLAT972_ALERT_2_C Check Calcd Resid. Dens.  0.96Ang From Ag01          -1.74 eA-3
PLAT972_ALERT_2_C Check Calcd Resid. Dens.  0.60Ang From N00M          -1.71 eA-3
PLAT972_ALERT_2_C Check Calcd Resid. Dens.  0.75Ang From Ag01          -1.71 eA-3
PLAT972_ALERT_2_C Check Calcd Resid. Dens.  0.86Ang From Ag02          -1.69 eA-3
PLAT972_ALERT_2_C Check Calcd Resid. Dens.  0.80Ang From Ag02          -1.67 eA-3
PLAT972_ALERT_2_C Check Calcd Resid. Dens.  0.71Ang From Ag03          -1.60 eA-3
PLAT972_ALERT_2_C Check Calcd Resid. Dens.  0.77Ang From Ag03          -1.57 eA-3
PLAT972_ALERT_2_C Check Calcd Resid. Dens.  0.62Ang From Ag03          -1.54 eA-3
PLAT977_ALERT_2_C Check Negative Difference Density on H017      .     -0.32 eA-3
PLAT977_ALERT_2_C Check Negative Difference Density on H01K      .     -0.37 eA-3
```

Alert level G

```
PLAT002_ALERT_2_G Number of Distance or Angle Restraints on AtSite          7 Note
PLAT003_ALERT_2_G Number of Uiso or U(i,j)  Restrained non-H-Atoms         11 Report
PLAT072_ALERT_2_G SHELXL First  Parameter in WGHT  Unusually Large       0.11 Report
PLAT083_ALERT_2_G SHELXL Second Parameter in WGHT  Unusually Large      24.71 Why ?
PLAT143_ALERT_4_G s.u. on c - Axis Small or Missing ..............    0.00040 Ang.
PLAT176_ALERT_4_G The CIF-Embedded .res File Contains SADI Records          3 Report
```

```
PLAT186_ALERT_4_G The CIF-Embedded .res File Contains ISOR Records          3 Report
PLAT242_ALERT_2_G Low    'MainMol' Ueq as Compared to Neighbors of       C016 Check
PLAT242_ALERT_2_G Low    'MainMol' Ueq as Compared to Neighbors of       C01A Check
PLAT242_ALERT_2_G Low    'MainMol' Ueq as Compared to Neighbors of       C01J Check
PLAT242_ALERT_2_G Low    'MainMol' Ueq as Compared to Neighbors of       C01Q Check
PLAT301_ALERT_3_G Main Residue  Disorder .............(Resd    1)          7% Note
PLAT434_ALERT_2_G Short Inter HL..HL Contact F008     ..F009     .       2.78 Ang.
                                             -1/2+x,y,1/2-z  =      6_556 Check
PLAT434_ALERT_2_G Short Inter HL..HL Contact F00C     ..F01U     .       2.72 Ang.
                                             3/2-x,-1/2+y,z  =      8_755 Check
PLAT720_ALERT_4_G Number of Unusual/Non-Standard Labels .........          89 Note
              Ag01    Ag02    Ag03    F004    F005    F006    F007    F008
              F009    F00A    F00B    F00C    F00D    F00E    F00F    F00G
              N00H    F00I    N00J    N00K    N00L    N00M    N00N    F00O
              C00P    H00P    C00Q    H00Q    C00R    C00S    C00T    C00U
              C00V    C00W    C00X    C00Y    C00Z    C010    C011    H011
              C012    C013    H013    C014    H014    C015    C016    C017
              H017    C018    C019    H019    C01A    C01B    C01C    H01C
              C01D    H01D    C01E    H01E    C01F    C01G    H01G    C01H
              C01I    C01J    C01K    H01K    C01L    H01L    C01M    H01M
              C01N    C01O    H01O    C01P    H01P    C01Q    C01R    H01R
              C01T    H01T    F01U    C01V    H01V    F01W    F01X    F01Y
              F01V
PLAT860_ALERT_3_G Number of Least-Squares Restraints ............          69 Note
PLAT883_ALERT_1_G Absent Datum for _atom_sites_solution_primary ..     Please Do !
PLAT910_ALERT_3_G Missing FCF Reflection(s) Below Theta(Min)[Deg]=       4.08 Note
                0  0  2,
PLAT912_ALERT_4_G Missing # of FCF Reflections Above STh/L=  0.600        237 Note
PLAT941_ALERT_3_G Average HKL Measurement Multiplicity ..........         3.9 Low
PLAT969_ALERT_5_G The 'Henn et al.' R-Factor-gap value ..........       2.885 Note
              Predicted wR2: Based on SigI**2  5.67 or SHELX Weight 15.59
PLAT978_ALERT_2_G Number C-C Bonds with Positive Residual Density.          0 Info
```

```
   0 ALERT level A = Most likely a serious problem - resolve or explain
   0 ALERT level B = A potentially serious problem, consider carefully
  30 ALERT level C = Check. Ensure it is not caused by an omission or oversight
  22 ALERT level G = General information/check it is not something unexpected

   1 ALERT type 1 CIF construction/syntax error, inconsistent or missing data
  37 ALERT type 2 Indicator that the structure model may be wrong or deficient
   8 ALERT type 3 Indicator that the structure quality may be low
   5 ALERT type 4 Improvement, methodology, query or suggestion
   1 ALERT type 5 Informative message, check
```

Validation response form

Please find below a validation response form (VRF) that can be filled in and pasted into your CIF.

```
# start Validation Reply Form
_vrf_PLAT213_lcz099
;
PROBLEM: Atom F00I          has ADP max/min Ratio .....        3.8 prolat
RESPONSE: ...
;
_vrf_PLAT342_lcz099
```

```
;
PROBLEM: Low Bond Precision on  C-C Bonds ..............    0.00872 Ang.
RESPONSE: ...
;
_vrf_PLAT410_lcz099
;
PROBLEM: Short Intra H...H Contact  H01K     ..H01R     .       1.95 Ang.
RESPONSE: ...
;
_vrf_PLAT906_lcz099
;
PROBLEM: Large K Value in the Analysis of Variance ......      4.384 Check
RESPONSE: ...
;
_vrf_PLAT911_lcz099
;
PROBLEM: Missing FCF Refl Between Thmin & STh/L=    0.600          6 Report
RESPONSE: ...
;
_vrf_PLAT934_lcz099
;
PROBLEM: Number of (Iobs-Icalc)/Sigma(W) > 10 Outliers ..          1 Check
RESPONSE: ...
;
_vrf_PLAT972_lcz099
;
PROBLEM: Check Calcd Resid. Dens.  0.81Ang From Ag01           -2.16 eA-3
RESPONSE: ...
;
_vrf_PLAT977_lcz099
;
PROBLEM: Check Negative Difference Density on H017      .      -0.32 eA-3
RESPONSE: ...
;
# end Validation Reply Form
```

It is advisable to attempt to resolve as many as possible of the alerts in all categories. Often the minor alerts point to easily fixed oversights, errors and omissions in your CIF or refinement strategy, so attention to these fine details can be worthwhile. It is up to the individual to critically assess their own results and, if necessary, seek expert advice.

PLATON version of 17/09/2025; check.def file version of 17/09/2025

---

duplicate check

No duplication found

Datablock lcz099 - ellipsoid plot

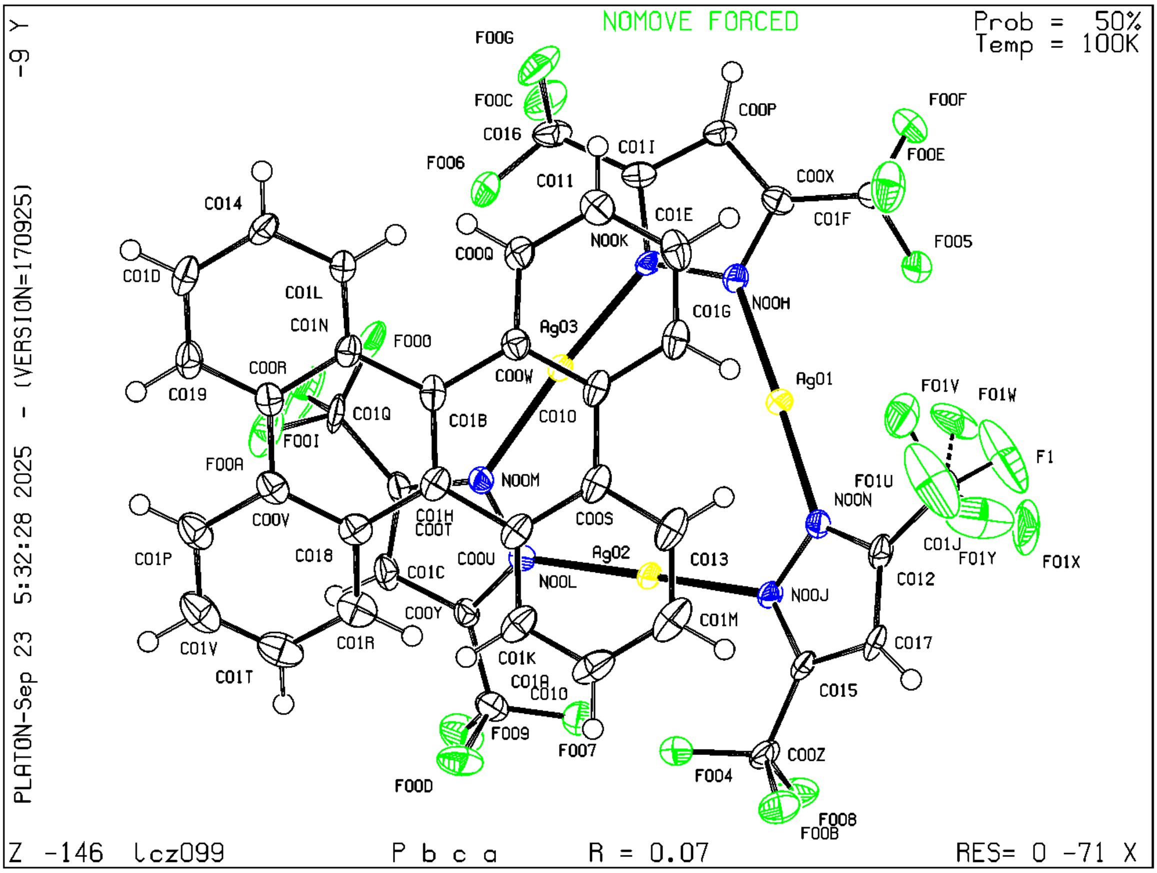

Supplement: Supplementary file 2 — Supporting File 2: anie72462‐sup‐0002‐DataFile.zip. [file ANIE-65-e1218503-s002.zip › supplementary_materials_crystallographic_data submit.zip!/cif_and_checkcif(1-114)/Ag3Pz3_99/Ag3Pz3_99_checkcif.pdf]
